# Ranging, activity budget, and diet composition of red titi monkeys (*Callicebus cupreus*) in primary forest and forest edge

**DOI:** 10.1007/s10329-015-0471-5

**Published:** 2015-05-21

**Authors:** Jenna Kulp, Eckhard W. Heymann

**Affiliations:** Behavioral Ecology and Sociobiology Unit, German Primate Center, Göttingen, Germany; Göttingen Centre for Biodiversity and Ecology, University of Göttingen, Göttingen, Germany; Fleischhauerstrasse 48, 23552 Lübeck, Germany

**Keywords:** Pitheciidae, Habitat use, Home range, Feeding, Secondary forest

## Abstract

Deforestation and fragmentation of tropical rainforests are increasingly creating forest edges and corresponding edge effects. Furthermore, primary forest is increasingly being replaced by secondary forest. The presence of high population densities of titi monkeys in fragmented and secondary forests suggests that they are capable of adapting to such habitat alterations. The aim of our study was to examine the ability of the red titi monkey (*Callicebus cupreus*) to adapt to forest edges and secondary forest. We compared home-range use, activity budgets, and diet composition in two groups of monkeys: one in primary forest and the other in primary forest with a long edge bordering secondary forest. The latter group avoided the secondary forest and used the edge in proportion to its availability. Groups did not differ in activity budgets but did show slight differences in diet composition. Taken together, our results suggest that there are no major effects of forest edges and secondary forest on red titi monkeys; however, given the relatively short study period, generalizations should be avoided until more comparative data become available. Furthermore, the age or successional stage of the secondary forest must be taken into consideration when drawing conclusions about its suitability as a primate habitat.

**Electronic supplementary material:**

The online version of this article (doi:10.1007/s10329-015-0471-5) contains supplementary material, which is available to authorized users.

## Introduction

Anthropogenic deforestation and forest fragmentation continue to threaten the huge biodiversity of tropical rainforests (Costa and Foley 2000; Negri et al. 2004). While many organisms disappear due to such changes, others persist but have to adjust to alterations in their habitats, particularly edge effects (Ries et al. 2004; Laurance et al. 2007). It is therefore important to know whether and how organisms deal with such effects in order to be able to predict their potential for long-term survival in altered habitats.

Primates may respond quite differently to forest disturbance, fragmentation, and edge effects, and the long-term survival of a particular primate species in an altered habitat depends on its specific habitat requirements (Bernstein et al. 1976; Schwarzkopf and Rylands 1989; Cowlishaw and Dunbar 2000). While *Procolobus rufomitratus*, for example, were found to be more likely to occupy fragments when the relative amount of habitat edge increased (Mbora and Meikle 2004), populations of *Procolobus pennantii* and *Colobus guereza* declined in fragmented forest (Chapman et al. 2007). *Cercopithecus mitis* and *Pan troglodytes* responded flexibly to anthropogenic habitat alteration leading to forest fragments within a cultivated landscape and modified their diet compositions (McLennan 2013; Tesfaye et al. 2013). Marsh (2003) suggested that smaller primate species are generally less affected by habitat fragmentation than larger species.

Similarly, edge effects may vary between species (Lidicker 1999). In Brazilian Amazonia, *Alouatta macconnelli*, *Chiropotes chiropotes*, *Saguinus midas*, and *Sapajus apella* showed higher population densities within 150 m of the edge compared to the forest interior, while the opposite was true for *Ateles paniscus* and *Pithecia chrysocephala* (Lenz et al. 2014). Groups of *Propithecus coquereli* living >1 km or <1 km from the edge did not differ in activity budget and food quality, but home ranges closer to the edge were more than twice as large as those further away (McGoogan 2011).

Titi monkeys (*Callicebus*) are a highly diverse genus of Neotropical primates (Ferrari et al. 2013). While some species have been considered habitat specialists (Kinzey and Gentry 1979; but see Defler 1994), the majority of species studied so far seem to show flexible habitat use and tolerate both natural and anthropogenic habitat disturbance (van Roosmalen et al. 2002; Bicca-Marques and Heymann 2013). Some persist or may even thrive in disturbed areas, secondary forest, and forest fragments (Ferrari et al. 2000; Heiduck 2002; van Roosmalen et al. 2002). *Callicebus ornatus* reach extremely high population densities in fragmented forests (Mason 1968; Wagner et al. 2009). This raises the question of how forest edges and secondary forest affect the ecology of titi monkeys. At forest edges and in young vegetation, leaf quality (the protein:fiber ratio) may increase due to higher light availability (Ganzhorn 1995). Also, insect abundance can be higher at the edges than in the interior of a forest (Fowler et al. 1993). The diet of titi monkeys is mainly based on fruit pulp and seeds, which are supplemented with variable amounts of leaves and invertebrates (Kinzey 1992; Norconk 2007; Heymann and Nadjafzadeh 2013). Therefore, we predicted that titi monkeys (1) prefer edge habitat over forest interior, (2) spend more time feeding in edge habitat, and (3) increase the proportion of leaves and/or (4) invertebrates in their diet in edge habitat.

At our study site in Peruvian Amazonia, a section of the forest was converted into a buffalo pasture in 1990, creating a long edge within the primary forest matrix. In 2001, the pasture was abandoned and has since been regenerating into secondary forest, but the edge remains clearly visible. This provided us with the opportunity to test the predictions stated above for *Callicebuscupreus*. To do this, we compared habitat use, activity patterns, and diet composition in two groups of *C. cupreus*, one living in the primary forest interior, the other bordering the forest edge. For the group bordering the edge, we examined whether time spent along the edge was higher than one would expect from its proportion of the home-range area, and we compared activity budget and diet composition between forest interior and edge.

## Methods

### Study site

The study was carried out at the Estación Biológica Quebrada Blanco (EBQB) in northeastern Peru, around 90 km south of Iquitos (4°21′S 73°09′W). This region has a humid tropical climate. Precipitation at the nearest meteorological station (Tamshiyacu, 4°00′S 73°09′W) averaged around 2700 mm/year (see Smith et al. 2004) and ranged between 2200 and 3500 mm/year in 2007–2010. Sunrise and sunset occurred around 0600 and 1800 h, respectively. For a detailed description of the study site, see Heymann (1995).

### Subjects

We investigated two habituated groups of *C. cupreus*. At the onset of observations, group 1 consisted of one adult male, one adult female, one subadult male, one juvenile male, and one carried infant, born late February. The infant started to locomote independently in June. The home range of this group included primary forest, forest edge, and secondary forest.

Group 2 consisted of three animals throughout the study: one adult male, one adult female, and one juvenile female. This group lived in primary forest with some small natural tree fall gaps and no access to the forest edge or secondary forest.

### Observational methods

The study was carried out from March to June 2011. Observations of groups alternated weekly, and each group was generally observed for six half-days (0600–1200 h, 1200–1800 h) per week, resulting in six statistical full days per month. In total, 69 h 40 min of focal animal sampling were performed for group 1 and 52 h 40 min for group 2. Activity data (defined in Table 1) were recorded continuously during 10-min focal animal samples (Martin and Bateson 2007), timing the onset and end of an activity with a stopwatch. Only independently locomoting animals were included in data collection (i.e., the infant in group 1 was only included in June). The order of focal animals was determined randomly, with at least 1 h left between separate observations of the same individual. When the focal animal was feeding, the food type was specified as pulp, pulp + seeds, leaf, flower, or prey. Differentiation between pulp and pulp + seeds was based on observations and on dropped feeding residuals; it was not possible to separate feeding on seeds only from pulp + seeds.

**Table 1:** Definition of activity categories

Activity	Definition (following Nadjafzadeh and Heymann 2008)
Feeding	Eating pulp, seeds, leaves, flowers, prey, or other food items
Foraging	Looking for food, holding and manipulating food, grabbing prey
Locomotion	Moving a distance of ≥1 m
Resting	Remaining stationary for at least 10 s without making body contact with another individual
Social	Remaining stationary with body contact; allogrooming; social playing; vocalizing
Other	Activities not fitting into any of the other categories, e.g., defecating, urinating

The locations of the study groups were measured at 15-min intervals with a Garmin GPSMap76CSx, as were the positions of all feeding plants, and the border between primary and secondary forest. We defined the forest edge as the 25-m strip from the border into the primary forest. We used 25 m as this corresponds approximately to the width of a gap created by a falling tree. Food plants were identified by Ricardo Zarate (Instituto de Investigaciones de la Amazonia Peruana, Iquitos).

### Data analysis

Home-range sizes were calculated as the 100 % minimum convex polygon (MCP) on the basis of GPS points in ArcGIS 9.3. The percentages of primary forest, forest edge, and secondary forest in the home range of group 1 were calculated for the 100 % MCP. These percentages represent the expected percentages of time spent in these habitats. We compared the observed percentage of time (= percentage of GPS points) spent in each habitat with the expected percentage using the *χ*^2^ test in Statistica 10.0.

In the data analyses we only included focal animal samples where the animal was visible for ≥5 min within the 10-min period. Only 2.6 % of the focal samples were incomplete for group 1, and 1.6 % for group 2. Times allocated to each activity were summed and expressed as percentages of the total focal sampling time to create activity budgets separately for groups 1 and 2, and separately for the primary forest and forest edge for group 1 (there were too few observations from secondary forest to calculate a meaningful activity budget). We compared activity budgets between groups 1 and 2 and between the primary forest and edge for group 1 using the *χ*^2^ test in Statistica 10.0.

To determine diet composition, we summed the time spent consuming different dietary items per group, and for group 1 per habitat type too, and expressed these times as percentages of the total feeding time. Because there were too many zero values for feeding in the secondary forest, statistical testing was not possible. We calculated the amount of time spent feeding per plant species, expressed this as a percentage of the total plant feeding time, and determined the top five plant species in the diets of groups 1 and 2. We calculated dietary diversity (Shannon index, *H*_s_ and *H*_max_), evenness (*E*), and the dietary overlap (Schoener index of overlap) between groups 1 and 2 using Microsoft Excel, after Lozán and Kausch (2007).

## Results

**Fig. 1:**
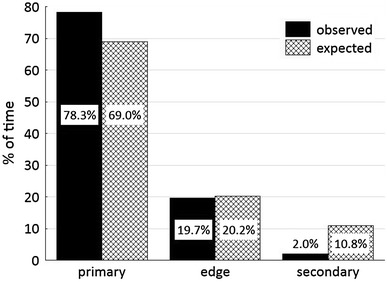
Observed and expected percentages of time spent by group 1 in the primary forest, forest edge, and secondary forest

Home-range size was 6.7 ha for group 1 and 11.4 ha for group 2 (Fig. A1 in the Electronic supplementary material, ESM). The observed habitat use of group 1 differed significantly from that expected (*χ*^2^ = 8.44, *df* = 2, *p* < 0.001), with more time spent in the primary forest and less time in the secondary forest than expected (Fig. 1). As group 1 avoided the secondary forest and used the edge in proportion to its availability, prediction 1 (preference for the edge) was not supported.

The prevailing activity of both groups was resting. Activity budgets did not differ between groups 1 and 2 (*χ*^2^ = 10.035, *df* = 5, n.s.; Table 2). The activity budget of group 1 did not differ between primary forest and forest edge (*χ*^2^ = 3.847, *df* = 5, n.s.; Table 2). Thus, prediction 2 (more time feeding on the edge than in the primary forest) was not supported.

**Table 2:** Activity budgets of groups 1 and 2

	Group 1			Group 2
	%	PF	FE	%
Feeding	10.4	10.5	10.4	16.1
Foraging	6.3	6.5	5.6	4.0
Locomotion	19.0	19.2	19.1	12.5
Resting	56.1	56.8	51.6	53.7
Social	8.0	6.8	13.3	13.4
Other	0.2	0.3	0.0	0.3

*PF* primary forest,* FE* forest edge

Fruit pulp was the principal dietary item in both groups (Table 3). Group 1 consumed pulp, pulp + seeds, and prey in the primary forest, almost exclusively pulp in the forest edge, and only prey in the secondary forest. For the reasons outlined above, did not perform a statistical test. Nevertheless, our data do not support predictions 3 and 4 (increases in leaf and prey consumption at the edge, respectively). Overall, 17 plant species were exploited by group 1 and 23 by group 2. The dietary diversity (Shannon index, *H*_s_ and *H*_max_) was marginally higher in group 2, while evenness (*E*) did not differ between the groups (group 1: *H*_s_ = 2.5, *H*_max_ = 2.9, *E* = 0.9; group 2: *H*_s_ = 2.9, *H*_max_ = 3.1, *E* = 0.9).

**Table 3:** Diet compositions of groups 1 and 2 (% of feeding time from focal animal sampling)

	Group 1				Group 2
	% (TFT: 6:47:10)	PF (TFT: 5:27:33)	FE (TFT: 1:19:25)	SF (TFT: 0:00:12)	% (TFT: 7:24:07)
Pulp	80.3	75.9	98.6	0.0	89.1
Pulp + seeds	11.4	14.2	0.0	0.0	0.0
Leaves	0.1	0.1	0.0	0.0	2.4
Prey	8.1	9.7	1.4	100.0	8.0
Other	0.0	0.0	0.0	0.0	0.5

*PF* primary forest, *FE* forest edge, *SF* secondary forest, *TFT* total feeding time (hours:minutes:seconds)

The diets of the groups overlapped strongly (Schoener index: 0.9). For both groups, the species most frequently consumed were *Oenocarpus bataua* (Arecaceae) and *Ophiocaryon klugii* (Sabiaceae) (Table A2 in the ESM).

## Discussion

The results of our study suggest that *C. cupreus* at the EBQB avoid secondary forest and do not increase the consumption of leaves or prey at the forest edge. The former observation is in accord with Heiduck’s (2002) finding that *Callicebus melanochir* preferred primary and avoided disturbed forest. However, it contrasts with many other reports which indicate that other *Callicebus* species are tolerant of secondary forest, or may even thrive and reach extremely high population densities in this habitat type (see the “Introduction”). A reason for these seemingly conflicting findings may arise from the successional stages of secondary forests. The secondary forest at EBQB is relatively young and structurally still very different from the primary forest, particularly with regard to vegetation cover and canopy height (Kupsch et al. 2014). It might simply be too young to represent a suitable habitat for *C. cupreus*. Tamarins at EBQB use the secondary forest seasonally (Culot et al. 2010; Kupsch et al. 2014). But, in contrast to claims that tamarins generally prefer secondary forest because of a higher abundance of arthropod prey (e.g., Terborgh 1983; Yoneda 1984; Rylands 1996), prey capture rates were lower (despite a higher abundance) and prey sizes were smaller in secondary compared to primary forest (Kupsch et al. 2014). This might be a consequence of a higher predation risk in the more open canopy of the secondary forest (Kupsch et al. 2014).

In a study of *Saimiri sciureus*, one group did not show seasonal variation in the use of early and late secondary forest, while a second group tended to use early secondary forest more frequently in the dry season (Stone 2007).

Before conclusions about primate preference for or avoidance of secondary forests can be drawn, information on forest age or successional stage as well as alternatives present in the habitat matrix is required. Macaques clearly preferred secondary forest imbedded in a matrix of *Acacia* plantations and secondary forest with agriculture (McShea et al. 2009). Some guenons, colobus monkeys, and mangabeys used secondary forest more than expected in a matrix of “mixed” forest, swamp forest, and forest dominated by a single tree species (Thomas 1991). In contrast, other species of guenons and colobus and one species each of mangabey, baboon, and chimpanzee used secondary forest much less than expected. Additionally, Thomas (1991) demonstrated interspecific differences in the use of tree fall gaps. While *Cercopithecus ascanius* showed a marked preference for gaps (for arthropod hunting), *C. mitis* and *Cercopithecus pogonias* did not show such a preference; they had a strong preference for large secondary forest compared to *C. ascanius*.

The forest edge was used by *C. cupreus* in proportion to its area. Our definition of forest edge is narrow and based on a criterion that can be easily operationalized in the field (25 m strip from the border into the primary forest). The diet composition of *C. cupreus* did not change at the forest edge, and predictions of dietary changes along the forest edge and in the secondary forest were not supported. However, we need to take into consideration the restricted study period (end of the rainy season and the start of the season with lower rainfall). To gain better insight into whether or not forest edges may affect the diet of *C. cupreus*, studies extending for longer periods with seasonal variations in food abundance are needed. At EBQB, fruit availability is low in the dry season (Knogge 1999), but some early-succession plants such as *Cecropia* or *Bellucia* fruit in the dry season and may attract *C. cupreus* into the secondary forest.

Finally, during our study, we increasingly got the impression that both study groups rested close to natural tree fall gaps. These are much smaller than the anthropogenic secondary forest, and edge effects may be completely different, not only due to the length of the edge but also due to the shape (long linear along the secondary forest, short and irregular along natural gaps). For future studies, it would be interesting to compare the use of such naturally disturbed areas with the areas next to the anthropogenically created secondary forest and its edge.

## Electronic supplementary material

Supplementary material 1 (DOC 197 kb)
