# Supplementary material for: Ranging, activity budget, and diet composition of red titi monkeys (Callicebus cupreus) in primary forest and forest edge
Source: Primates. 2015 May 21;56(3):273–8. doi: 10.1007/s10329-015-0471-5 (PMC4473277; doi:10.1007/s10329-015-0471-5)

Supplementary material

Table A1. Home-range size (Minimum Convex Polygon) of Groups 1 and 2, percentage of primary forest, edge, and secondary forest in the home range of Group 1, and number and percentage of GPS points take at 15-min intervals

|  | Group 1 | | | | Group 2 |
| --- | --- | --- | --- | --- | --- |
|  | Home-range size | % Primary forest | %  Edge | % Secondary forest | Home-range size |
| Area [ha] | 6.7 | 4.6 | 1.4 | 0.7 | 11.4 |
| % of area |  | 69.0 | 20.2 | 10.8 |  |
| # GPS points | 639 | 500 | 126 | 13 | 563 |
| % GPS points | 100.0 | 78.3 | 19.7 | 2.0 | 100.0 |

Table A2. Top five plant species in the diet of Groups 1 and 2

|  | Group 1 | | Group 2 | |
| --- | --- | --- | --- | --- |
| Rank | Plant species  (plant family) | % in diet | Plant species  (plant family) | % in diet |
| 1 | *Oenocarpus bataua*  (Arecaceae) | 24.5 | *Oenocarpus bataua*  (Arecaceae) | 15.4 |
| 2 | *Ophiocaryon klugii*  (Sabiaceae) | 16.7 | *Ophiocaryon klugii*  (Sabiaceae) | 14.6 |
| 3 | *Paullinia* sp. 1  (Sapindaceae) | 11.1 | *Naucleopsis* sp.  (Moraceae) | 14.4 |
| 4 | *Couma macrocarpa*  (Apocynaceae) | 8.1 | *Inga* sp. 1  (Fabaceae) | 9.6 |
| 5 | *Iryanthera paraensis*  (Myristicaceae) | 7.6 | *Licania* sp. 1  (Chrysobalanaceae) | 9.1 |
|  | Σ top 5 | 68.0 | Σ top 5 | 63.1 |

Figure A1. Map of the study area with home ranges of Group 1 (violet polygon) and Group 2 (yellow polygon), the primary forest (dark green), the secondary forest (light green). The area between the orange line and the secondary forest represents the edge forest. The small white area within the home range of Group 1 represents the camp clearing; blue lines are creeks. Black lines show the trail system.


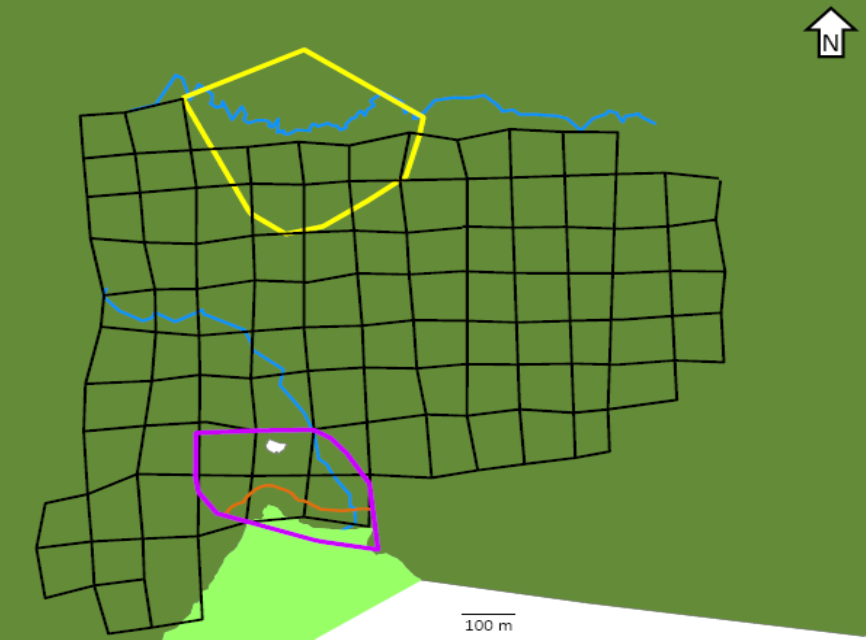

Supplement: Supplementary file 1 — Supplementary material 1 (DOC 197 kb) [file 10329_2015_471_MOESM1_ESM.doc]
